# Taxonomic and Functional Shifts in the Perinatal Gut Microbiome of Rhesus Macaques

**DOI:** 10.1128/spectrum.00814-22

**Published:** 2022-07-11

**Authors:** Nicholas S. Rhoades, Isaac R. Cinco, Sara M. Hendrickson, Mark K. Slifka, Ilhem Messaoudi

**Affiliations:** a Department of Microbiology, Immunology and Molecular Genetics, University of Kentucky, College of Medicine, Lexington, Kentucky, USA; b Division of Neuroscience, Oregon National Primate Research Center, Oregon Health & Sciences University, Beaverton, Oregon, USA; University of Nebraska—Lincoln

**Keywords:** pregnancy, microbiome, NHP, perinatal, rhesus macaque, gut microbiome, metagenomics

## Abstract

Pregnancy and the postpartum period result in some of the most dramatic metabolic, hormonal, and physiological changes that can be experienced by an otherwise healthy adult. The timing and magnitude of these changes is key for both maternal and fetal health. One of the factors believed to critically modulate these physiological changes is the maternal gut microbiome. However, the dynamic changes in this community during the perinatal period remain understudied. Clinical studies can be complicated by confounding variables like diet and other drivers of heterogeneity in the human microbiome. Therefore, in this study, we conducted a longitudinal analysis of the fecal microbiome obtained during the pregnancy and postpartum periods in 26 captive rhesus macaques using 16S rRNA gene amplicon sequencing and shotgun metagenomics. Shifts at both the taxonomic and functional potential level were detected when comparing pregnancy to postpartum samples. Taxonomically, Alloprevotella, Actinobacillus, and Anaerovibrio were enriched in the gut microbiome during pregnancy, while Treponema, *Lachnospiraceae*, and Methanosphaera were more abundant postpartum. Functionally, the gut microbiome during pregnancy was associated with increased abundance in pathways involving the production of the short-chain fatty acid (SCFA) butyrate, while pathways associated with starch degradation and folate transformation were more abundant during the postpartum period. These data demonstrate dramatic changes in the maternal gut microbiome even in the absence of dietary changes and suggest that rhesus macaques could provide a valuable model to determine how changes in the microbiome correlate to other physiological changes in pregnancy.

**IMPORTANCE** Pregnancy and the postpartum period are characterized by a myriad of metabolic and physiological adaptations needed to support fetal growth and maternal health. The maternal gut microbiome is believed to play a key role during this period but remains underexplored. Here, we report significant shifts in the taxonomic landscape and functional potential of the gut microbiome in 26 pregnant rhesus macaques during the transition from pregnancy to the postpartum period, despite shared dietary and environmental exposures. Increased abundance of pathways involved in the production of the short-chain fatty acid butyrate could play a critical role in modulating the maternal immune system and regulating fetal tolerance. On the other hand, increased abundance of pathways associated with starch degradation and folate transformation during the postpartum period could be important for meeting the metabolic demands of breastfeeding and neonatal growth.

## INTRODUCTION

The perinatal period, which encompasses pregnancy up to 1 year postpartum, is characterized by large physiological, immunological, and hormonal changes that impact maternal and fetal health. This shift is characterized by an increase in insulin resistance, along with increased levels of leptin and adiponectin (1, 2), which are critical to ensure that the fetus receives adequate nutrition and prepare the mother for metabolic demands imposed by lactation. A disruption of these metabolic adaptations can lead to adverse outcomes like gestational diabetes and preeclampsia (3–5), which in turn can have a negative impact on the infant, including high incidence of preterm birth and increased incidences of infection, large for gestational age (LGA), and reduced cognitive development (6–8). One of the factors that significantly modulates maternal metabolism but remains understudied during the perinatal period is the gut microbiome (9).

The gut is home to the most densely populated microbial community in the human body, composed of bacterial symbionts and commensals, as well as archaeal, fungal, and viral members (10). The gut microbiome plays critical roles in vitamin production (11), immune homeostasis (12), and metabolism of indigestible substrates, among other functions (13). This community stabilizes in adulthood (14) but remains sensitive to environmental factors like dietary shifts (15) and antibiotic use (16), as well as other shifts in host physiology, such as pregnancy (17). Previous studies found that the gut microbiome during pregnancy was less diverse and more variable than that of healthy nonpregnant individuals and harbored an increased abundance of *Proteobacteria* (18). However, more recent studies found that the microbiome remained stable during pregnancy but shifted significantly in the postpartum period (19). Additionally, stress during pregnancy can exacerbate this dysbiotic microbiome state (20), which has also been implicated in postpartum depression (21).

Another key reason for understanding the dynamics of the perinatal maternal gut microbiome is the vital role it plays in seeding the infant microbiome (22). The infant is first exposed to maternal vaginal and fecal microbes at birth. The development and maintenance of the infant gut microbiome has long-term ramifications for the maturation of the immune system (23), protection from enteric infection (24), and establishment of a healthy metabolic state (25). The establishment of the infant microbiome is influenced by a multitude of factors, such as delivery method (26), antibiotic use (27), and breastfeeding (28).

Rhesus macaques are a valuable preclinical model to study the role of the microbiome in health and disease, as they have a gut microbiome similar to that of humans, especially those in the developing world (29, 30). Moreover, rhesus macaques are a vital model for the study of perinatal and reproductive health (31, 32). In this study, we utilized a combination of 16S rRNA amplicon and shotgun metagenomic sequencing to longitudinally characterize the fecal microbiome of rhesus macaques during the perinatal period. Specifically, we investigated longitudinal changes in the taxonomic composition and functional potential of the maternal gut microbiome during pregnancy and the postpartum period. We observed both taxonomic and functional shifts within the fecal microbiome within individual animals and across our entire study population before and after delivery. Taxonomically, Alloprevotella, Actinobacillus, and Anaerovibrio were enriched during pregnancy, while Treponema, *Lachnospiraceae*, and Methanosphaera were more abundant during the postpartum period. Functionally, pregnancy was associated with an increased abundance of pathways producing butyrate, a short-chain fatty acid (SCFA) that is beneficial during pregnancy, while the postpartum period was characterized by increased abundance of pathways associated with starch degradation and folate transformation.

## RESULTS

### Taxonomic shifts in the perinatal gut microbiome.

**FIG 1:**
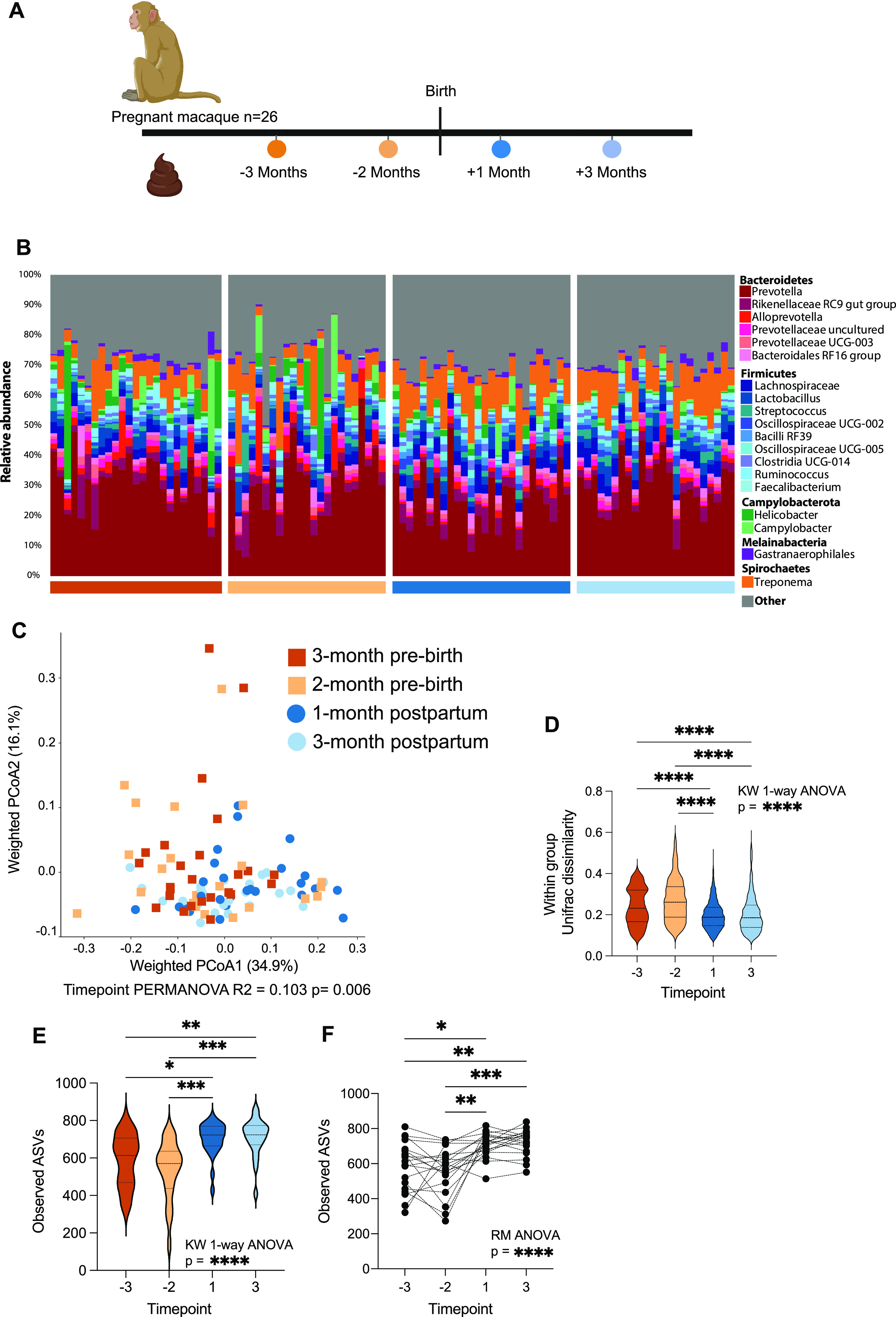
Landscape and perinatal shifts of the maternal gut microbiome. (A) Study design. (B) Stacked bar plot organized by time point. All taxa below 1% average abundance were grouped into the “Other” category. Each vertical bar represents a single sample. (C) Principal coordinate analysis (PCoA) of weighted UniFrac distances between microbial communities colored by time point. (D) Violin plot of weighted UniFrac distances between the fecal microbiome samples collected at the same time point. (E) Violin plot of observed amplicon sequencing variants (ASVs) at each time point. Horizontal lines within each violin indicate the median value along with the 25th and 75th percentiles for that time point. Significance for data in panels D and E was determined using Kruskal-Wallis (KW) 1-way nonparametric ANOVA with Dunn’s *post hoc* test. *, *P* < 0.05; **, *P* < 0.01; ***, *P* < 0.001; ****, *P* < 0.0001. (F) Scatterplot of observed ASVs across time points with lines connecting samples collected from the same individual. Each dot represents an individual sample, with solid lines connecting samples from the same individual across time. Significance for data in panel F was determined using nonparametric one-way repeated-measure ANOVA (Friedman test) with Dunn’s *post hoc* comparisons between time points. Horizontal lines above the plot denote significance of *post hoc* tests. *, *P* < 0.05; **, *P* < 0.01; ***, *P* < 0.001.

We utilized 16S rRNA gene amplicon sequencing of rectal swab samples collected prebirth during pregnancy (~90 and 60 days prior to birth) and the postpartum period (~30 and 90 days after giving birth) (Fig. 1A shows the experimental design) to determine shifts in microbial communities during the perinatal period. Prior to analysis, we confirmed that samples were free of PCR contamination and bias by using negative controls and sequenced community standards (Fig. S1A in the supplemental material). Across all samples, the rhesus macaque gut microbiome was dominated by *Bacteroidetes* (Prevotella, *Rikenellaceae*, and Alloprevotella) and *Firmicutes* (*Lachnospiraceae*, Lactobacillus, and Streptococcus), along with *Proteobacteria* (Helicobacter and Campylobacter) and *Spirochetes* (Treponema) (Fig. 1B). Despite the lack of changes in housing or diet of the studied rhesus macaques, we observed a distinct shift in the overall composition of the maternal gut microbiome when comparing samples collected during pregnancy and postpartum (Fig. 1C, time point permutational multivariate analysis of variance [PERMANOVA] *R*^2^ = 0.103, *P* = 0.006). At both time points measured during pregnancy, the maternal gut microbiome was more variable than the gut microbiome during the postpartum period (Fig. 1D, Kruskal Wallis nonparametric [KW] analysis of variance [ANOVA], *P* < 0.0001, and Dunn’s *post hoc* comparison between pre- and postpartum time points, *P* < 0.0001, as noted on the graph). Additionally, samples collected postpartum harbored a greater number of observed amplicon sequence variants (ASVs) than samples collected during pregnancy (Fig. 1E, KW ANOVA, *P* < 0.0001, and Dunn’s *post hoc* comparison between pregnancy and postpartum time points, *P* < 0.05 to 0.001, as noted on the graph). This pattern was observed across the entire cohort and when we conducted a pairwise comparison for animals that had samples from all four time points (Fig. 1F, repeated measures [RM] ANOVA, *P* < 0.0001, and Dunn’s *post hoc* comparison between pregnancy and postpartum time points, *P* < 0.05 to 0.001, as noted on the graph).

**FIG 2:**
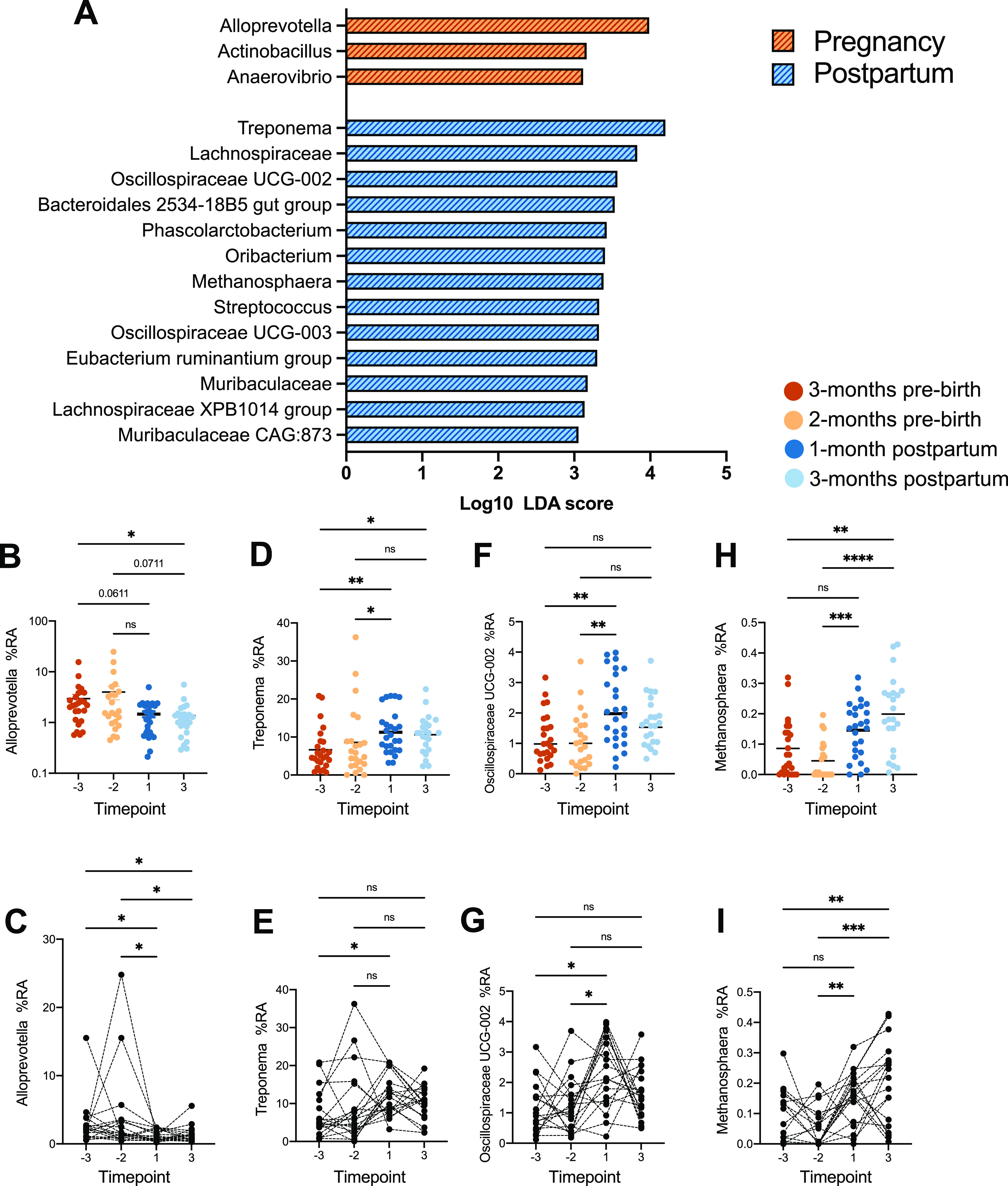
Taxonomic drivers of perinatal gut microbiome shifts. (A) Taxa that were differentially abundant between pregnant and postpartum samples, with pregnancy versus postpartum used as the subject and individual time points as the class. Differential abundance was determined using LEfSe (Log_10_ LDA score of >2). (B to I) Scatterplots of abundance data for Alloprevotella (B, C), Treponema (D, E), *Oscillospiraceae* UCG-002 (F, G), and Methanosphaera (H, I). RA, relative abundance. In panels B, D, F, and H, each dot represents an individual sample, colored by time point. Significance of the data was measured by nonparametric one-way repeated-measure ANOVA (Friedman test) with Dunn’s *post hoc* comparisons between time points. Asterisks denote significance of *post hoc* tests as follows: *, *P* < 0.05; **, *P* < 0.01; ***, *P* < 0.001; ****, *P* < 0.0001; ns, not significant. In panels C, E, G, and I, each dot represents an individual sample, with solid lines connecting samples from the same individual across time. Significance of the data was determined using 1-way ANOVA with the *post hoc* Šidàk multiple-comparison test. **, *P* < 0.01; ***, *P* < 0.001; ns, not significant.

Furthermore, we also compared pregnancy and postpartum samples using linear discriminant analysis effect size (LEfSe) to determine which taxa were driving the observed differences in gut microbiome composition between pregnancy and postpartum samples. During pregnancy, the maternal gut microbiome was enriched in Alloprevotella, Actinobacillus, and Anaerovibrio (Fig. 2A, Table S2), while the gut microbiome postpartum was enriched in Treponema, multiple *Lachnospiraceae*, and Methanosphaera, among others (Fig. 2A, Table S2). The enrichment of Alloprevotella in early pregnancy samples was driven by a significantly higher abundance of this taxon in both preterm samples compared to its abundance in postpartum samples, a trend that remained when conducting pairwise comparisons (Fig. 2B and C). On the other hand, the relative abundance of Treponema was lower during pregnancy than postpartum (Fig. 2D and E). While the shifts in these higher-abundance genera were significant, we observed more dramatic shifts in the abundances of less-abundant taxa postpartum. For example, *Oscillospiraceae* UCG-002 was transiently more abundant 1 month postpartum, while Methanosphaera increased at both postpartum time points (Fig. 2F to I). Many of these taxonomic trends across the entire study population were consistent with pairwise analysis conducted on animals that had samples collected across all time points (Fig. 2C, E, G, and I).

### Comparison of the perinatal gut microbiome to that of nongravid individuals.

To determine how these changes relate to the nongravid gut microbiome, we compared our findings to those reported for healthy, age-matched, nongravid (e.g., nonpregnant) female macaques living in the same primate center and sampled during the same year (33). The overall composition of the nongravid microbiome was distinct from that of the perinatal gut microbiome at both time points (Fig. S1B, time point PERMANOVA, *R*^2^ = 0.218, *P* = 0.001). Furthermore, the number of ASVs observed was found to increase significantly during the postpartum period compared to the numbers of ASVs observed at the nongravid and pregnant time points and did not change when only comparing the two latter groups (Fig. S1C, KW ANOVA, *P* < 0.0001, and Dunn’s *post hoc* comparison between pregnancy and postpartum time points, *P* < 0.01 to 0.001, as noted on the graph). We next compared the compositions of the nongravid, pregnancy, and postpartum communities using LEfSe to determine which taxa were driving the observed differences in gut microbiome composition. The gut microbiome of nongravid female macaques was enriched in Methanobrevibacter, along with multiple Eubacterium and *Oscillospiraceae* species, relative to those of pregnant and postpartum females (Fig. S1D). The gut microbiome of pregnant females was instead enriched in Alloprevotella, Actinobacillus, and Anaerovibrio relative to that of nongravid females (Fig. S1D). Finally, in comparison to the pregnant state, several Treponema species were more abundant in the postpartum gut microbiome (Fig. S1D and S1E). Interestingly, although the levels of Methanobrevibacter increased postpartum, they did not reach nongravid levels by 3 months (Fig. S1E). Similarly, the relative abundance of Alloprevotella increased throughout pregnancy but failed to return to nongravid levels during the postpartum period (Fig. S1E). The levels of Treponema, Marvinbryantia, Lachnospira, Fibrobacter, Faecalibacterium, and Dorea, among others, increased with pregnancy and remained elevated up to 3 months postpartum (Fig. S1E).

### Shotgun metagenomics reveal both taxonomic and functional shifts in the gut microbiome during the perinatal period.

**FIG 3:**
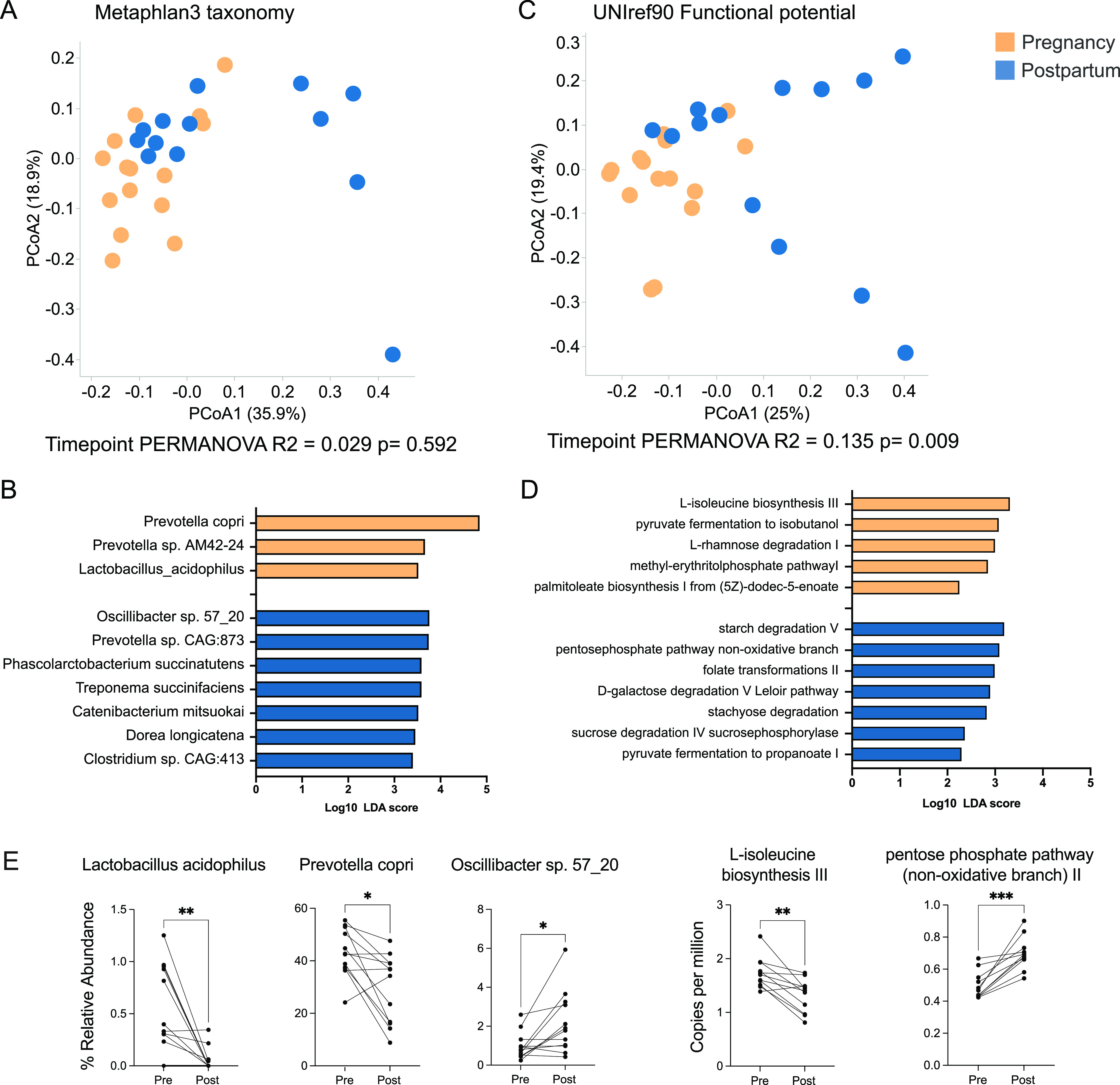
Perinatal shifts in the functional potential and species-level taxonomy of the maternal microbiome. (A) Principal coordinate analysis (PCoA) of Bray-Curtis dissimilarity built on species-level abundance from MetaPhlan3 and colored by time point. (B) Species that were differentially abundant between pre- and postbirth samples (LEfSe, Log_10_ LDA score of >2). (C) PCoA of Bray-Curtis dissimilarity built on the abundances of all functional genes annotated using HUMAnN3 and the Uniref90 database and colored by time point. (D) MetaCyc pathways that were differentially abundant between pregnancy and postpartum samples (LEfSe, Log_10_ LDA score of >2). (E) Pairwise scatterplots of select differentially abundant bacterial species and functional pathways for animals that had data generated at both time points. Dots represent individual samples, with each solid line connecting samples from the same animal across time. Significance was determined using a nonparametric Wilcoxon matched-pairs signed-rank test. *, *P* < 0.05; **, *P* < 0.001; ***, *P* < 0.0001.

To further explore shifts in the metabolic potential of the gut microbiome during the perinatal period, we utilized shotgun metagenomics. Shotgun metagenomic libraries were prepared from a subset of fecal samples collected 2 months prior to birth and 1 month postpartum (*n* = 15/time point). Two postpartum libraries had less than 1 million reads after host decontamination and were excluded from future analysis. In contrast to the 16S rRNA gene amplicon sequencing data, the overall taxonomic compositions assessed by shotgun metagenomics did not differ significantly between the pregnancy and postpartum samples, based on Bray-Curtis dissimilarity (Fig. 3A, time point PERMANOVA, *R*^2^ = 0.029, *P* = 0.592). This disagreement could be due to lower sample numbers used for our shotgun metagenomic experiment or, potentially, driven by the lack of phylogenetically informed beta diversity metrics (UniFrac) for shotgun metagenomic data. Nevertheless, we were able to identify multiple differentially abundant species between these two time points (Table S3). For example, the abundances of Prevotella species, the most abundant genera from our 16S data, shifted from an enrichment of Prevotella copri and Prevotella sp. AM42-24 in prebirth samples to Prevotella sp. CAG:873 in postpartum samples (Fig. 3B). Species that were enriched in postpartum samples relative to their abundances in prebirth samples included Oscillibacter sp. 57_20, Phascolarctobacterium succinatutens, and Treponema succinifaciens (Fig. 3B).

We also used shotgun metagenomics to assess the functional potential of the gut microbiome in pregnant rhesus macaques and found that the overall functional potentials differed between pregnancy and postpartum samples (Fig. 3C, time point PERMANOVA, *R*^2^ = 0.135, *P* = 0.009). In total, 77 MetaCyc metabolic pathways were differentially abundant between the two time points (Table S4). More specifically, the functional capacity of the late-pregnancy microbiome showed enrichment of “pyruvate fermentation to isobutanol,” “methyl-erythritol phosphate pathway,” and “L-isoleucine biosynthesis” pathways (Fig. 3D). In contrast, the early-postpartum gut microbiome had higher abundances of pathways involved in “starch degradation,” “folate transformation,” and “pyruvate fermentation to propionate” (Fig. 3D). Many of the differentially abundant species and pathways remained significant when conducting nested pairwise analysis using data from animals that had samples from both time points (Fig. 3E, Fig. S2).

**FIG 4:**
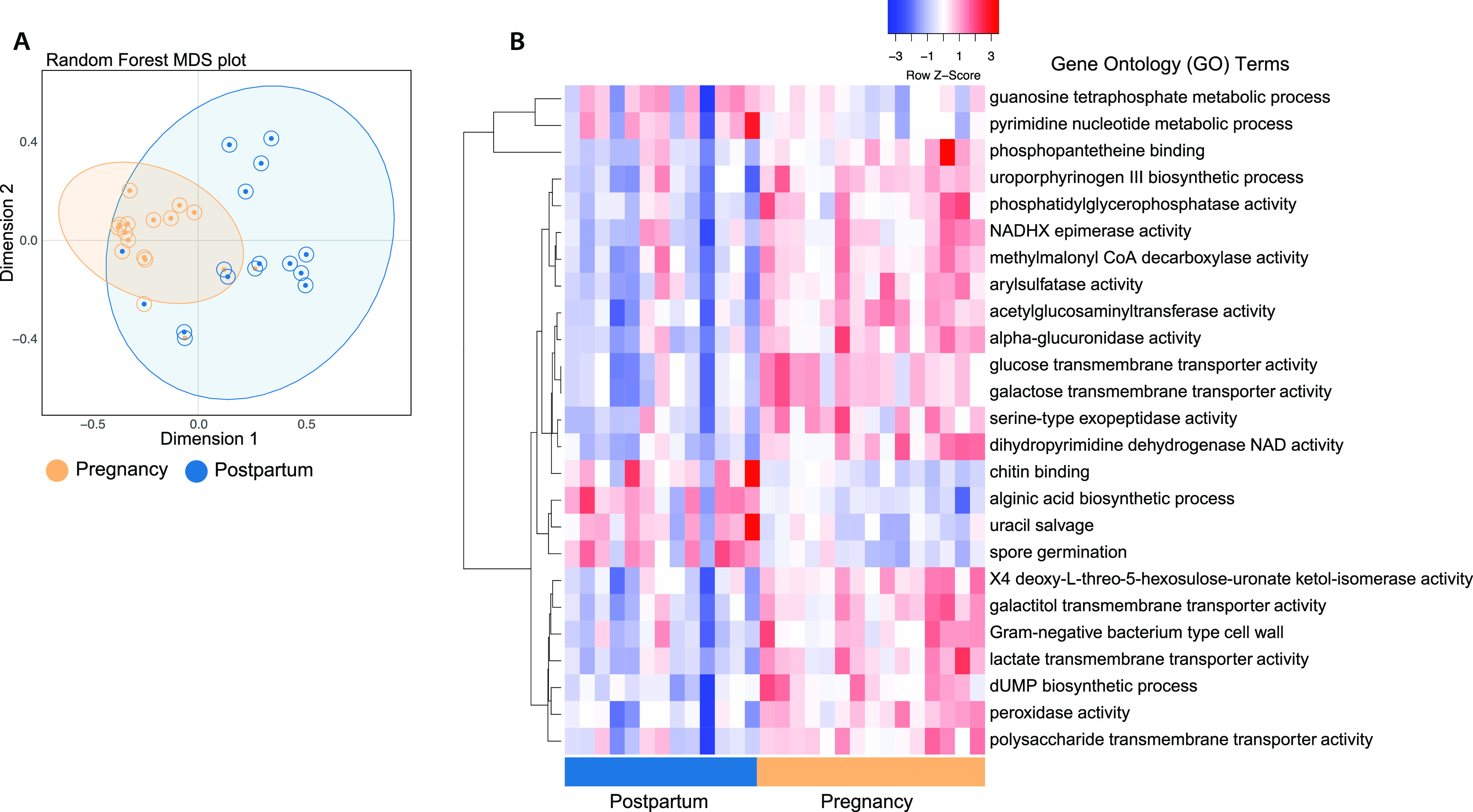
Random forest analysis highlights functional shifts in the perinatal gut microbiome. (A) Random forest multidimensional scaling (MDS) plot of proximity scores from a random forest analysis of Gene Ontology (GO) terms, colored by time point. For each data point, the color of the dot denotes the sample’s actual group. The color of the ring around each dot represents which group that sample was classified in. Any sample that was misclassified has a mismatched dot and surrounding ring. (B) Heat map of the 25 most statistically significant GO terms as predicted by random forest modeling.

To better understand which microbial functions best distinguished the pre- and postdelivery time points, we used a supervised random forest model built using Gene Ontology (GO) terms, which annotate gene products based on their molecular functions, the biological processes they are involved in, and the cellular locations where they are found. The overall random forest model was 82% accurate at classifying samples into the two time points (Fig. 4A). Next, we extracted and plotted the abundances of the 25 GO terms that best distinguished the two time points (Fig. 4B). Many of the GO terms that were more abundant in pregnancy samples were associated with metabolism and transport of carbohydrates, notably “alpha glucuronidase activity,” “galactose transmembrane transporter activity,” and “lactate transmembrane transporter activity” (Fig. 4B). Interestingly, some of the GO terms that best distinguished postpartum samples were associated with fungi rather than bacteria (e.g., “spore germination” and “chitin binding”) (Fig. 4B).

## DISCUSSION

The perinatal period encompasses some of the most dramatic shifts in metabolism, immunity, and hormonal balance that a healthy adult can experience. It is well established that the gut microbiome can modulate these shifts. Indeed, a recent study of a cohort in rural Zimbabwe found that the taxonomic composition of the maternal fecal microbiome was strongly predicted by gestational age, birth weight, and neonatal growth (34). However, our understanding of the changes that the maternal gut microbiome undergoes during the perinatal period is limited. In this study, we explored taxonomic and functional shifts in the gut microbiome of rhesus macaques during the perinatal period, with particular emphasis on pregnancy versus postpartum microbiomes. One limitation of this study is that prepregnancy and early-pregnancy samples (1st trimester) were not collected, which precluded us from fully capturing the dynamics of the changes in the maternal gut microbiome.

To address this limitation, we carried out a cross-sectional analysis and compared the microbial communities detected during pregnancy and the postpartum period to those of healthy nongravid females living in the same facility and surveyed during the same window of time using the same experimental approaches (data set extracted from reference 33). This comparison indicated a significant impact of pregnancy on the maternal microbiome. Our data indicate that the maternal microbiome was less rich (lower numbers of observed ASVs) but more heterogeneous (increased within-group dissimilarity [beta diversity]) during pregnancy than during the postpartum period. However, the diversity of the gut microbiome ~3 months postpartum was still reduced relative to that of nongravid animals. This increase in beta diversity with pregnancy is in line with data from multiple human and animal studies, particularly late in pregnancy (17–19, 35). On the other hand, previous clinical studies have reported a drop, an increase, or no change in the alpha diversity of the maternal microbiome (17, 18, 36), likely due to the high heterogeneity within the healthy human microbiome (37).

The relative abundances of several Treponema species and Alloprevotella were increased in the gut microbiome during the perinatal period compared to their abundances in nongravid animals. These taxa are uncommon in the human gut microbiome in the developed world but common in the gut microbiome of humans from low- and middle-income countries, especially those living in a rural rather than urban setting (38, 39). These taxa are also highly prevalent in the rhesus macaque gut microbiome. The increased abundances of these taxa during the perinatal period are in line with the recent study from Zimbabwe (34), further highlighting the utility of the rhesus macaque in the study of gut microbiomes from non-Western societies (29, 40). The increased abundance of these fiber-degrading bacteria may play a critical role in meeting the energy demands of pregnancy and breastfeeding.

We also observed a depletion of Faecalibacterium, as previously reported (18). Additionally, the loss of the methanogenic Methanobrevibacter is also observed in healthy pregnant women but not individuals with pregravid obesity (41). Intriguingly, previous studies in humans have reported an enrichment in *Actinobacteria*, especially Bifidobacterium, a key commensal in the infant microbiome, during late pregnancy (42). Additionally, increased abundances of *Firmicutes* and *Proteobacteria* and decreased abundance of *Bacteroidetes* during late and postpregnancy were also reported (18, 19). We observed no significant changes in the abundances of taxa belonging to *Actinobacteria* or *Proteobacteria* during the postpartum period, despite reporting Bifidobacterium colonization in infant macaques and humans (29). However, we did observe increases in multiple *Firmicutes* postpartum, including *Lachnospiraceae*, Phascolarctobacterium, *Oscillospiraceae*, and Eubacterium, among others. Many of these taxa are associated with the production of beneficial SCFA within the gut (43, 44), which is linked to lower blood pressure and reduced preeclampsia risk in humans (45, 46).

Many of the perinatal taxonomic trends we observed at both the 16S rRNA amplicon and shotgun metagenomic level were driven by taxa with unique metabolic capacities, such as the methanogenic Methanosphaera and fiber-degrading Treponema, thus suggesting that the metabolic capacity of the prenatal gut microbiome changes during the perinatal period. Many of our findings, including the increased potential to produce butyrate in late pregnancy, agree with what has been observed in humans (47). Additionally, pathways associated with the production of the monosaturated fatty acid palmitoleate were increased in the microbiome of pregnant individuals. Palmitoleate has been shown to play an important role in the maintenance of placental trophoblasts by preventing apoptosis (48). We also observed increased enrichment of GO terms associated with the transportation of simple sugars like lactate, glucose, and galactose in late pregnancy compared to the GO terms in postpartum samples. This increase in the potential for simple sugar transport in the gut microbiome could indicate that more of these metabolites are reaching the large intestine during pregnancy. Whether this shift is due to increased caloric intake or a change in host absorption is unclear.

During the postpartum period, the gut microbiome of rhesus macaques was enriched in pathways like starch degradation, folate transformation, and stachyose degradation, which are important for the degradation of complex substrates and production of secondary metabolites rather than the metabolism of simple sugars observed in late pregnancy. This switch is likely a reflection of the host nutritional needs. Additionally, our random forest analysis revealed that the post-birth gut microbiome was enriched in GO terms associated with fungal spore germination and colonization in the gut. To our knowledge, the gut mycobiome during the perinatal period has not been examined previously. However, fungal components like chitin have been shown to be an important and potentially community-modulating carbon source in the gut (49). In our data, we found increased enrichment of pathways associated with chitin binding in the postpartum period. Degradation of this component can improve the epithelial barrier and alter cytokine production (50, 51).

## MATERIALS AND METHODS

### Cohort description.

Longitudinal rectal swab samples were obtained at 4 time points from a total of 26 reproductive-age female rhesus macaques, 19 of which had a sample collected at every time point (Table S1). These time points were 3 months prebirth (90.2 ± 20.5 days prebirth [mean ± standard deviation]), 2 months prebirth (60.0 ± 20.0 days prebirth), 1 month postbirth (29.8 ± 1.5 days postbirth), and 3 months postbirth (91.2 ± 4.0 days postbirth). The rhesus macaques were housed in outdoor small-group shelters at the Oregon National Primate Research Center (ONPRC). All rhesus macaque studies were overseen and approved by the OHSU/ONPRC Institutional Animal Care and Use Committees (IACUCs) according to the National Institutes of Health’s *Guide for the Care and Use of Laboratory Animals* (52). Animals were housed according to the standards established by the U.S. Federal Animal Welfare Act (53) and the *Guide for the Care and Use of Laboratory Animals* (52). All animals were tested for simian viruses (simian immunodeficiency virus, simian retrovirus 2, macacine herpesvirus 1, and simian T lymphotropic virus) and received a tuberculin test semiannually. All animals were vaccinated against Campylobacter coli at both pregnancy time points for an unrelated study. The rhesus macaques in this study were fed twice daily with Lab Diet, Monkey Diet 5038 (Ralston Purina, St. Louis, MO, USA). This diet is guaranteed to contain no more than 15% crude protein, 5% crude fat, 6% crude fiber, 9% ash, and 12% moisture. This diet is supplemented with seasonal fresh fruit and produce once daily. Municipal water is available *ad libitum*.

### 16S rRNA amplicon sequencing.

Total DNA was extracted from rectal swabs using the DNeasy PowerSoil pro kit (Qiagen, Valencia, CA, USA). The hypervariable V4-V5 region of the 16S rRNA gene was amplified using PCR primers (515F/926R with the forward primers, including a 12-bp barcode). PCRs were conducted in duplicate and contained 12.5 μL GoTaq master mix, 9.5 μL nuclease-free H_2_O, 1 μL template DNA, and 1 μL 10 μM primer mix. The thermal cycling parameters were 94°C for 5 min, 35 cycles of 94°C for 20 s, 50°C for 20 s, 72°C for 30 s, followed by 72°C for 5 min. PCR products were purified using a MinElute 96 UF PCR purification kit (Qiagen, Valencia, CA, USA). Libraries were sequenced (2 × 300 bases) using an Illumina MiSeq.

Raw FASTQ 16S rRNA gene amplicon sequences were uploaded and processed using the QIIME2 analysis pipeline (54). Briefly, sequences were demultiplexed and then quality filtered using DADA2 (55), which filters chimeric sequences and generates an amplicon sequence variant (ASV) table equivalent to an operational taxonomic unit (OTU) table at 100% sequence similarity. Sequence variants were then aligned using MAFFT (56), and a phylogenetic tree was constructed using FastTree2 (57). Taxonomy was assigned to sequence variants using q2-feature-classifier against the SILVA database (release 138) (58). To prevent sequencing depth bias, samples were rarified to 13,781 sequences per sample before alpha and beta diversity analysis. This depth was selected based on the maximum depth that included all samples. QIIME 2 was also used to calculate ASV richness, a metric of alpha diversity. Beta diversity was estimated in QIIME 2 using weighted and unweighted UniFrac distances (59).

### Shotgun metagenomic library preparation and analysis.

Shotgun metagenomic libraries were prepared from a subset of fecal samples collected 2 months prior to birth and 1 month postpartum (*n* = 15/time point) using the iGenomx Riptide (iGenomx, South San Francisco, CA) according to iGenomx’s recommended protocol and sequenced on an Illumina HiSeq 4000 (2 × 100). Raw demultiplexed reads were quality filtered using Trimmomatic (parameters of ILLUMINACLIP, small_RNA_trim_PE.fa:2:30:10; LEADING, 3; TRAILING, 3; SLIDINGWINDOW, 4:15; MINLEN, 36) (60), and potential host reads were removed by aligning trimmed reads to the Macaca mulatta genome (Mmul 8.0.1) using BowTie2 (61). Trimmed and decontaminated reads were then annotated using the HUMAnN3 pipeline with default settings against the UniRef90 database. UniRef90 gene families were assigned to MetaCyc pathways and mapped to Gene Ontology (GO) terms. The abundances of MetaCyc pathways and GO terms were normalized using copies per million (CPM) prior to statistical analysis (62–64). Species-level taxonomy was assigned to quality-controlled short reads using Metaphlan3 (65). Bray-Curtis dissimilarity matrices were constructed for both species-level relative abundance (MetaPhlan3) and normalized gene annotations (UniRef90) using the vegdist function in the R package Vegan (66). Principal coordinate analysis (PCoA) was conducted using the base R function cmdscale.

### Statistics.

To determine whether the gut microbiome during pregnancy was distinct from the microbiome during the postpartum period, we ran PERMANOVAs on weighted UniFrac distances for 16S rRNA amplicon data and Bray-Curtis distances for both taxonomic and functional shotgun metagenomic data using the function ADONIS from the R package Vegan (66). For single-factor comparisons across more than two time points, such as ASV richness or relative abundance of a specific taxon, we performed 1-way, nonparametric Kruskal-Wallis ANOVAs with Dunn’s *post hoc* test in PRISM (version 8). For pairwise comparisons of more than two time points, nonparametric one-way repeated-measure ANOVA (Friedman test) with the Šidàk multiple-comparison test was used, while the nonparametric Wilcoxon matched-pairs signed-rank test was utilized for two-group pairwise comparisons. The linear discriminant analysis effect size (LEfSe) algorithm was used to identify taxa and pathways that were differentially abundant between pregnancy and postpartum samples, with a logarithmic linear discriminant analysis (LDA) score cutoff of 2 (67). For the 2-time-point comparisons, time point was used as the class for the LEfSe comparison, whereas for the 4-time-point comparisons, prebirth versus postpartum was used as the class and time point as the subclass. A random forest analysis was performed to determine which GO terms best predicted pre- and postdelivery time points, using the R package rfPermute. The z-scores for each of the top 25 GO terms were calculated and displayed as a heatmap using the heatmap.2 function in the R package gplot.

### Data availability.

16S rRNA gene and shotgun metagenomic sequencing data are deposited in the National Center for Biotechnology Information (NCBI) sequence read archive (SRA) under the BioProject accession number PRJNA816841. The 16S rRNA gene data from the nongravid samples used can be found under accession number PRJNA800766.
