# Supplementary material for: Taxonomic and Functional Shifts in the Perinatal Gut Microbiome of Rhesus Macaques
Source: Microbiol Spectr. 2022 Jul 11;10(4):e00814-22. doi: 10.1128/spectrum.00814-22 (PMC9431225; doi:10.1128/spectrum.00814-22)
Supplement: Supplemental file 1 — Supplemental material. Download spectrum.00814-22-s001.pdf, PDF file, 0.5 MB [file spectrum.00814-22-s001.pdf]

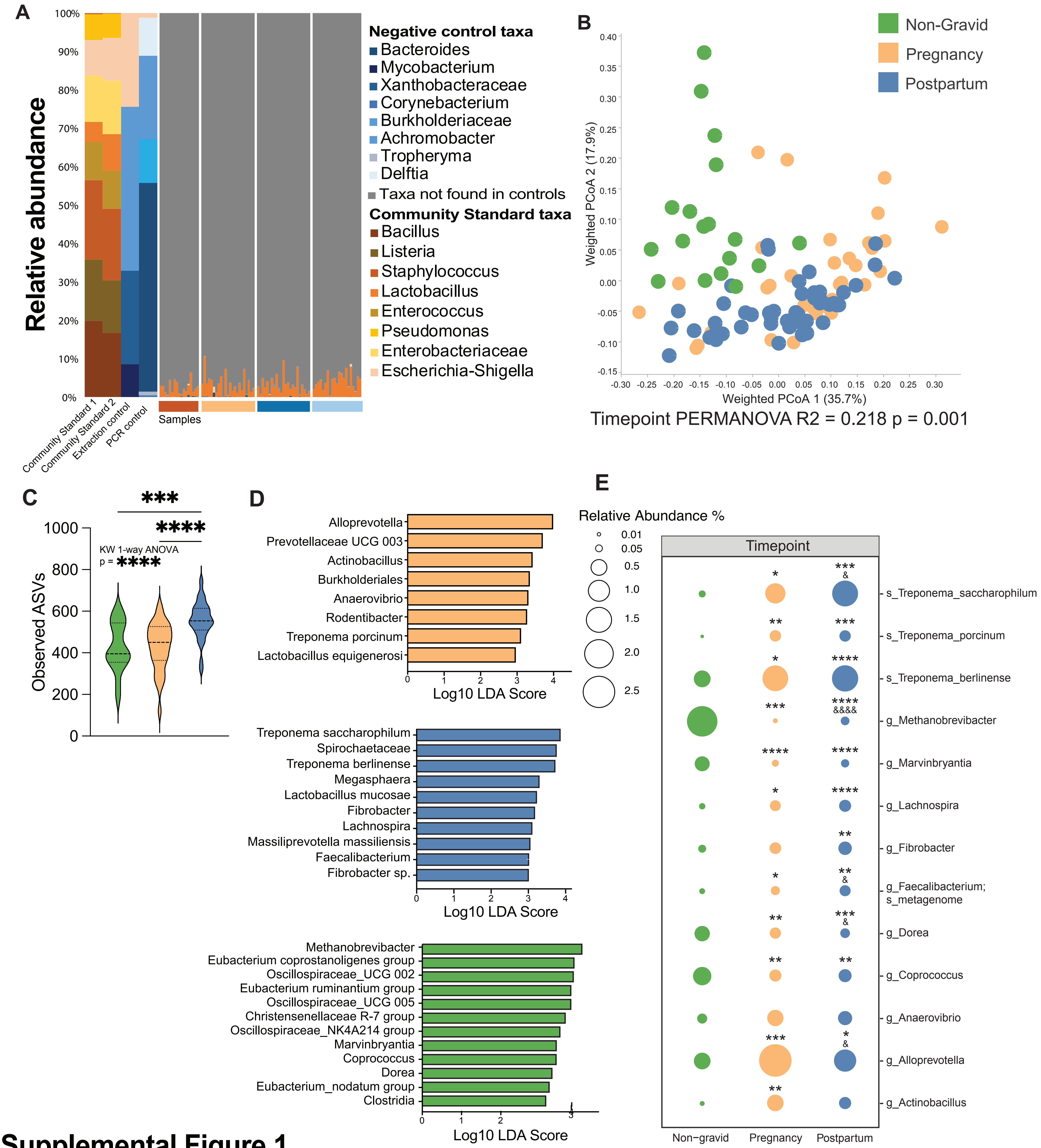

Supplemental Figure 1

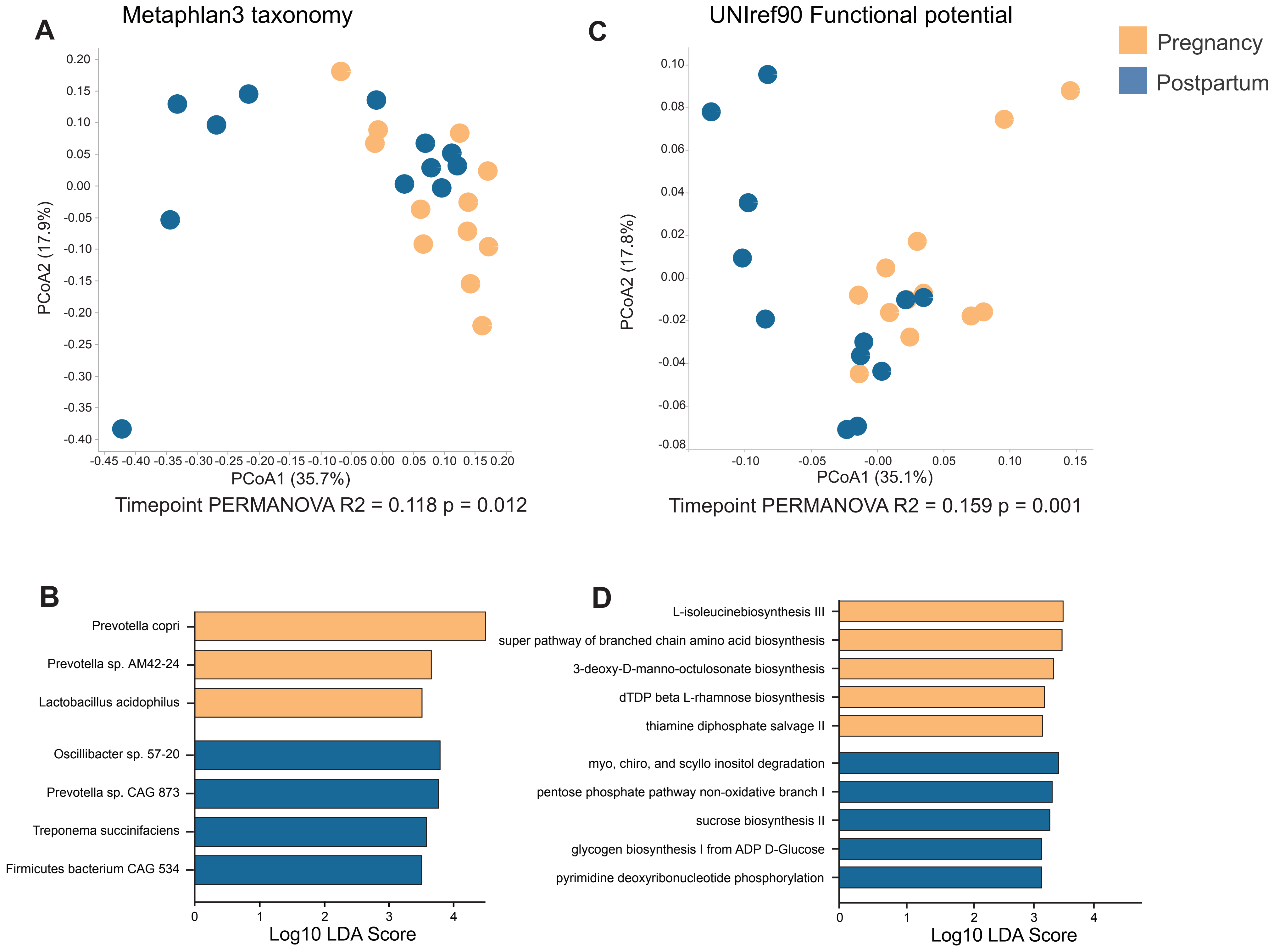

Supplemental Figure 2

Figure S1: **Additional taxonomic and pairwise comparisons.** (A) Stacked bar plot comparing microbial communities of control samples to the biological samples included in this study. (B) PCoA of weighted UniFrac distances between fecal, microbial communities by timepoint. (C) Violin plots of amplicon sequence variants (ASVs) for each timepoint. (D) Differentially abundant taxa by timepoint with non-gravid vs. pregnancy vs. postpartum used as subject and time-point as class determined via LEfSe (Log10 LDA Score > 2). (E) Bubble plot of different taxa sequenced at each time-point, with the % relative abundance (%RA) of each being represented by the size of its bubble. Significance for panel C was determined using Kruskal-Wallis (KW) 1-way non-parametric ANOVA with Dunn's post-hoc-test * $p < 0.05$, ** $p < 0.01$, *** $p < 0.001$, **** $p < 0.0001$. Significance for panel E was determined using a 2-way ANOVA with post-hoc Tukey multiple comparisons test, * $p < 0.05$, ** $p < 0.01$, *** $p < 0.001$, **** $p < 0.0001$ when comparing pregnancy and postpartum vs. non-gravid and & $p < 0.05$, && $p < 0.01$, &&& $p < 0.001$, &&&& $p < 0.0001$ when comparing pregnancy vs. postpartum.

Figure S2: Pairwise data recapitulates the perinatal shifts in the functional potential and species level taxonomy of the maternal microbiome seen in the larger dataset. (A) PCoA of Bray-Curtis dissimilarity built on species-level abundance from MetaPhlan3. Colored by timepoint and including only animals with pairwise data. (B) Differentially abundant species between pregnancy and postpartum samples including only animals with pairwise data (LEfSe, Log_{10} LDA score > 2). (C) PcoA of Bray-Curtis dissimilarity built on the abundance of all functional genes annotated using

HUMAnN 3 and the Uniref90 database. Colored by timepoint and including only animals with pairwise data. (D) Differentially abundant Metacyc pathways between pregnancy and postpartum samples including only animals with pairwise data. (LEfSe, Log_{10} LDA score > 2).

ID	Age (y)	Parity	Pre-birth 16S Sample 1	Pre-birth 16S Sample 2	Date gave Birth	Post-birth 16S Sample 1	Post-birth 16S Sample 2	Pre-birth Shotgun	Post-birth Shotgun	Notes
Dam_1	18.4	8	2/15/17	NA	7/9/17	8/9/17	10/10/17	No	No	
Dam_2	13.2	7	2/13/17	3/13/17	5/23/17	6/23/17	8/23/17	yes	yes	
Dam_3	10.1	7	2/15/17	3/15/17	5/15/17	6/12/17	8/14/17	yes	yes	
Dam_4	9.1	3	2/17/17	3/17/17	5/5/17	6/2/17	8/2/17	No	No	
Dam_5	8.7	5	2/15/17	NA	6/1/17	6/30/17	8/28/17	yes	yes	
Dam_6	8	5	NA	3/15/17	6/6/17	7/5/17	9/5/17	yes	yes	
Dam_7	7	5	2/17/17	3/17/17	6/3/17	7/5/17	9/5/17	yes	yes	
Dam_8	7	5	2/15/17	3/15/17	5/15/17	6/12/17	8/14/17	yes	yes	
Dam_9	7	5	2/17/17	3/17/17	6/10/17	7/12/17	NA	yes	yes	
Dam_10	6.8	4	2/17/17	3/17/17	4/29/17	5/30/17	7/27/17	No	No	
Dam_12	6	2	2/17/17	3/17/17	NA	NA	NA	yes	No	Fetal loss towards end of pregnancy
Dam_11	6	3	2/17/17	3/17/17	6/20/17	7/18/17	9/15/17	No	No	
Dam_13	5.8	4	2/13/17	3/13/17	4/13/17	5/12/17	7/12/17	NA	yes	
Dam_14	6	4	2/13/17	3/13/17	6/5/17	7/5/17	9/5/17	yes	yes	
Dam_15	6	4	2/13/17	3/13/17	6/20/17	7/18/17	9/15/17	yes	yes	
Dam_16	5.1	4	2/13/17	3/13/17	5/20/17	6/19/17	8/18/17	yes	yes	
Dam_17	5.2	2	2/13/17	3/13/17	5/30/17	6/30/17	NA	yes	yes	
Dam_18	5.1	3	2/13/17	3/13/17	6/15/17	7/18/17	9/15/17	No	No	
Dam_19	5.1	2	2/13/17	3/13/17	4/11/17	5/12/17	7/12/17	No	No	
Dam_20	5.2	4	2/13/17	NA	6/19/17	7/18/17	9/15/17	yes	yes	
Dam_21	4.1	1	2/13/17	3/13/17	6/7/17	7/5/17	9/5/17	yes	yes	
Dam_22	4.1	3	2/13/17	3/13/17	5/3/17	6/2/17	8/2/17	No	No	
Dam_23	4.1	2	2/13/17	3/13/17	5/19/17	6/19/17	8/18/17	No	No	
Dam_24	3.9	1	2/13/17	3/13/17	5/11/17	6/9/17	8/9/17	No	No	
Dam_25	3.8	2	2/13/17	3/13/17	4/24/17	5/22/17	NA	No	No	baby lost 39 days post birth - unknown causes
Dam_26	3.8	1	2/13/17	3/13/17	5/23/17	6/23/17	8/23/17	No	No	
Adult_1	8	NA								
Adult_2	8.1	NA								
Adult_3	8.1	NA								
Adult_4	8.1	NA								
Adult_6	8.4	NA								
Adult_8	8.8	NA								
Adult_9	9	NA								
Adult_10	9.1	NA								
Adult_11	7.9	NA								
Adult_12	7.9	NA								
Adult_13	7.7	NA								
Adult_14	8.5	NA								
Adult_15	7.7	NA								
Adult_16	9.9	NA								
Adult_17	8.8	NA								
Adult_18	9.2	NA								
Adult_19	7	NA								
Adult_20	7	NA								

Supplemental Table 1: Cohort Characteristics

Total L6 taxa in analysis = 78
Total differentially abundant L6 taxa = 16

Taxa	Timepoint	LDA Score	P-Value
Muribaculaceae_g__CAG_873	post	3.057431108	1.81E-05
Lachnospiraceae_XPB1014_group	post	3.139222077	4.10E-05
Muribaculaceae	post	3.178036936	0.000196765
Eubacterium__ruminantium_group	post	3.305622785	0.000220507
Oscillospiraceae_g__UCG_003	post	3.327854247	7.96E-06
Streptococcus	post	3.330337161	0.000926788
Methanosphaera	post	3.386605446	5.39E-07
Oribacterium	post	3.406137493	2.36E-05
Phascolarctobacterium	post	3.429017025	2.07E-05
Bacteroidales p_2534_18B5_gut_group	post	3.53384377	0.000379446
Oscillospiraceae_g__UCG_002	post	3.571338776	0.000345141
Lachnospiraceae___	post	3.830000632	2.14E-05
Treponema	post	4.200587581	0.000160882
Anaerovibrio	pre	3.119192881	0.005976681
Actinobacillus	pre	3.164600663	0.005905506
Alloprevotella	pre	3.98882586	0.000814164

Supplemental Table 2: LEfSe results of genera that are differentially abundant in post- and pre-birth samples.

Total species in analysis = 88
Total differentially abundant species = 29

Species	Timepoint	LDA Score	P-Value
Firmicutes_bacterium_CAG_95	Post	3.02383749	0.01519015
Dorea_formicigenerans	Post	3.02980546	0.01198325
Holdemanella_biformis	Post	3.07401633	0.00371993
Firmicutes_bacterium_CAG_555	Post	3.08840736	0.0038812
Firmicutes_bacterium_CAG_238	Post	3.09310629	0.01260648
Blastocystis_sp_subtype_1	Post	3.12585674	0.01826078
Campylobacter_coli	Post	3.12860649	0.00629978
Roseburia_hominis	Post	3.13243459	0.00184124
Ruminococcus_sp_CAG_563	Post	3.14629454	0.03006716
Firmicutes_bacterium_CAG_170	Post	3.20685201	0.00734694
Eubacterium_hallii	Post	3.20989414	0.00998219
Coprococcus_comes	Post	3.22052019	0.0052956
Oscillibacter_sp_CAG_241	Post	3.23357699	0.00074084
Fibrobacter_intestinalis	Post	3.28432256	0.00140642
Firmicutes_bacterium_CAG_791	Post	3.2877569	0.0114055
Firmicutes_bacterium_CAG_534	Post	3.32514089	0.00340535
Ruminococcus_sp_CAG_488	Post	3.37238993	0.0015651
Collinsella_aerofaciens	Post	3.38691156	0.02449882
Eubacterium_siraeum	Post	3.39473917	0.03306762
Clostridium_sp_CAG_413	Post	3.39759472	0.01204391
Dorea_longicatena	Post	3.45231283	0.00891629
Catenibacterium_mitsuokai	Post	3.51789463	0.0486098
Treponema_succinifaciens	Post	3.58560388	0.01765908
Phascolarctobacterium_succinatutens	Post	3.58591476	0.01681373
Prevotella_sp_CAG_873	Post	3.74247251	0.0060994
Oscillibacter_sp_57_20	Post	3.75782793	0.02000256
Lactobacillus_acidophilus	Pre	3.518122	0.00027784
Prevotella_sp_AM42_24	Pre	3.66390143	0.01558762
Prevotella_copri	Pre	4.84629164	0.00255054

Supplemental Table 3: LEfSe results of species that are differentially abundant in post- and pre-birth samples.

Total pathways in analysis = 282
Total differentially abundant pathways = 77

Pathway	Timepoint	LDA Score	P-value
PWY_7237_myo__chiro_andscillo_inositoldegradation	Post	3.25095377	0.01146901
PWY_6737_starchdegradationV	Post	3.19051619	0.00344291
ARGSYNBSUB_PWY_L_argininebiosynthesisII_acetylcycle_	Post	3.11010295	0.00025006
NONOXIPENT_PWY_pentosephosphatepathway_non_oxidativebranch_	Post	3.09055472	2.50E-05
PWY_7187_pyrimidinedeoxyribonucleotidesdenovobiosynthesisII	Post	3.07798848	0.01372061
PWY0_162_superpathwayofpyrimidineribonucleotidesdenovobiosynthesis	Post	3.05626505	0.04034605
CALVIN_PWY_Calvin_Benson_Basshamcycle	Post	3.05113003	0.00011984
ARGSYN_PWY_L_argininebiosynthesisI_viaL_ornithine_	Post	3.03468271	0.00020869
PWY_7400_L_argininebiosynthesisIV_archaebacteria_	Post	3.02638376	0.00017381
PWY0_166_superpathwayofpyrimidinedeoxyribonucleotidesdenovobiosynthesis_E_coli_	Post	2.99772032	0.02259378
PWY_3841_folatetransformationsII	Post	2.99097204	0.03219039
TRNA_CHARGING_PWY_tRNAcharging	Post	2.98491227	0.02000256
PWY_7197_pyrimidinedeoxyribonucleotidephosphorylation	Post	2.90930631	0.03608457
PWY66_422_D_galactosedegradationV_Leloirpathway_	Post	2.89450167	0.00344291
PWY_6317_galactosedegradationI_Leloirpathway_	Post	2.89258379	0.00296624
PWY_4981_L_prolinebiosynthesisII_fromarginine_	Post	2.88672509	0.00070492
PWY_6121_5_aminoimidazoleribonucleotidebiosynthesisI	Post	2.87308697	0.01056926
HISDEG_PWY_L_histidinedegradationI	Post	2.8725249	0.01056926
GLUTORN_PWY_L_ornithinebiosynthesis	Post	2.84741653	0.00807883
PWY_6527_stachyosedegradation	Post	2.82434145	0.00807883
GOLPDLCAT_PWY_superpathwayofglyceroldegradationto1_3_propanediol	Post	2.75637375	0.02319342
PWY_724_superpathwayofL_lysine_L_threonineandL_methioninebiosynthesisII	Post	2.73093047	0.01558762
PWY_7007_methylketonebiosynthesis	Post	2.63172304	0.01409334
P185_PWY_formaldehydeassimilationIII_dihydroxyacetonecycle_	Post	2.62010534	0.00969837
PWY0_1061_superpathwayofL_alaninebiosynthesis	Post	2.53448788	0.00158575
PWY_5101_L_isoleucinebiosynthesisII	Post	2.47471479	0.00459869
P164_PWY_purinenucleobasesdegradationI_anaerobic_	Post	2.41103401	0.01625822
PWY_621_sucrosedegradationIII_sucroseinvertase_	Post	2.4031445	0.01423078
COLANSYN_PWY_colanicacidbuildingblocksbiosynthesis	Post	2.38671204	0.0083581
POLYAMINSYN3_PWY_superpathwayofpolyaminebiosynthesisII	Post	2.38009131	0.02127292
PWY_5384_sucrosedegradationIV_sucrosephosphorylase_	Post	2.36322108	0.03011819
PWY_1861_formaldehydeassimilationII_RuMPCycle_	Post	2.3586077	0.00147505
PWY_7323_superpathwayofGDP_mannose_derivedO_antigenbuildingblocksbiosynthesis	Post	2.35676927	0.007258
GLYCOCAT_PWY_glycogendegradationI_bacterial_	Post	2.32403254	0.01826078
ARGININE_SYN4_PWY_L_ornithinedenovobiosynthesis	Post	2.30933492	0.00969837
PWY_7115_C4photosyntheticcarbonassimilationcycle_NAD_MEtype	Post	2.29618947	0.01101525
P108_PWY_pyruvatefermentationtopropanoateI	Post	2.29570365	0.01046324
PWY_7328_superpathwayofUDP_glucose_derivedO_antigenbuildingblocksbiosynthesis	Post	2.29541968	0.04321127
ARGORNPROST_PWY_arginine_ornithineandprolineinterconversion	Post	2.29170117	0.01122961
P161_PWY_acetylenedegradation	Post	2.18069689	0.01741195
PWY4LZ_257_superpathwayoffermentation_Chlamydomonasreinhardtii_	Post	2.17604062	0.01321162
SALVADEHYPOX_PWY_adenosinenucleotidesdegradationII	Post	2.14012582	0.04265153
P125_PWY_superpathwayof_R_R__butanediolbiosynthesis	Post	2.06544079	0.03306762
PWY_6608_guanosinenucleotidesdegradationIII	Post	2.04503889	0.03805624
PWY_5103_L_isoleucinebiosynthesisIII	Pre	3.31085428	0.00035689
PWY_5695_uratebiosynthesis_inosine5_phosphatedegradation	Pre	3.29602543	1.34E-05
BRANCHED_CHAIN_AA_SYN_PWY_superpathwayofbranchedaminoacidbiosynthesis	Pre	3.29160958	0.00035689
RIBOSYN2_PWY_flavinbiosynthesisI_bacteriaandplants_	Pre	3.25409673	0.00020869
PWY_6151_S_adenosyl_L_methioninecycleI	Pre	3.24990683	0.0007097
PWY_1269_CMP_3_deoxy_D_manno_octulosonatebiosynthesisI	Pre	3.19191108	0.00014447
PWY_7219_adenosineribonucleotidesdenovobiosynthesis	Pre	3.15783556	0.00083858
ASPASN_PWY_superpathwayofL_aspartateandL_asparaginebiosynthesis	Pre	3.11268476	0.00083858
ILEUSYN_PWY_L_isoleucinebiosynthesisI_fromthreonine_	Pre	3.08053068	0.00187461
VALSYN_PWY_L_valinebiosynthesis	Pre	3.08053068	0.00187461
PWY_7111_pyruvatefermentationtoisobutanol_engineered_	Pre	3.07678809	0.00296624
PWY_6897_thiaminsalvageII	Pre	3.06971015	0.00059944
DTDPRHAMSYN_PWY_dTDP_L_rhamnosebiosynthesisI	Pre	3.06690448	0.01205394
PWY_7221_guanosineribonucleotidesdenovobiosynthesis	Pre	3.05103804	0.01558762
PWY_2942_L_lysinebiosynthesisIII	Pre	3.02614493	0.0011638
PEPTIDOGLYCANSYN_PWY_peptidoglycanbiosynthesisI_meso_diaminopimelatecontaining_	Pre	3.01226665	0.00136694
PWY_6387_UDP_N_acetylmuramoyl_pentapeptidebiosynthesisI_meso_diaminopimelatecontaining_	Pre	3.01050677	0.00296624
RHAMCAT_PWY_L_rhamnosedegradationI	Pre	2.99898731	0.00083858
PWY_6386_UDP_N_acetylmuramoyl_pentapeptidebiosynthesisII_lysine_containing_	Pre	2.9750093	0.00160236
PWY_7357_thiaminformationfrompyrithiamineandoxythiamine_yeast_	Pre	2.9524079	0.00059944
PWY_6385_peptidoglycanbiosynthesisIII_mycobacteria_	Pre	2.93075704	0.00344291
PWY_5097_L_lysinebiosynthesisVI	Pre	2.8759834	0.00461107
COA_PWY_coenzymeAbiosynthesisI	Pre	2.87312348	0.04037343
NONMEVIPP_PWY_methylerythritolphosphatepathwayI	Pre	2.85069912	0.00029847
THISYNARA_PWY_superpathwayofthiamindiphosphatebiosynthesisIII_eukaryotes_	Pre	2.82712907	0.0007097
PWY_7560_methylerythritolphosphatepathwayII	Pre	2.7972022	6.74E-05
PWY_6163_chorismatebiosynthesisfrom3_dehydroquinate	Pre	2.76286408	0.00704261
PWY_7539_6_hydroxymethyl_dihydropterindiphosphatebiosynthesisIII_Chlamydia_	Pre	2.65963191	0.04508694
PWY_6147_6_hydroxymethyl_dihydropterindiphosphatebiosynthesisI	Pre	2.65379744	0.04508694
PWY_6270_isoprenebiosynthesisI	Pre	2.62948117	0.00035623
PWYG_321_mycolatebiosynthesis	Pre	2.3868696	0.02539156
PWY0_862__5Z__dodec_5_enoatebiosynthesis	Pre	2.31021118	0.04508694
PWY_6282_palmitoleatebiosynthesisI_from_5Z__dodec_5_enoate_	Pre	2.25247495	0.04034605

Supplemental Table 4: LEfSe results of pathways that are differentially abundant in post- and pre-birth samples.
